# Remotely prescribed and monitored home-based gait-and-balance therapeutic exergaming using augmented reality (AR) glasses: protocol for a clinical feasibility study in people with Parkinson’s disease

**DOI:** 10.1186/s40814-024-01480-w

**Published:** 2024-03-27

**Authors:** L. E. S. Hardeman, D. J. Geerse, E. M. Hoogendoorn, J. Nonnekes, M. Roerdink

**Affiliations:** 1 Department of Human Movement Sciences, Faculty of Behavioural and Movement Sciences, Vrije Universiteit Amsterdam, Amsterdam Movement Sciences, Amsterdam, The Netherlands; 2 Department of Rehabilitation, Centre of Expertise for Parkinson & Movement Disorders, Donders Institute for Brain, Cognition and Behaviour, Radboud University Medical Centre, Nijmegen, The Netherlands; 3 Department of Rehabilitation, Sint Maartenskliniek, Nijmegen, The Netherlands

**Keywords:** Parkinson’s disease, Augmented-reality, Clinical feasibility, Home-based therapy, Rehabilitation, Exergaming, Remote monitoring, Gait, Balance

## Abstract

**Background:**

Clinical guidelines for people with Parkinson’s disease (pwPD) stress that, complementary to pharmacological treatment, exercise and physiotherapy should be given a central role in disease management. Adhering to regular exercise of the right type, and with high repetition, remains a challenge for pwPD. Exergaming has the potential to increase adherence through play and personalised interventions, both in clinic and at home. Reality DTx^®^ is an augmented-reality (AR) home-based gait-and-balance exergaming intervention specifically designed for pwPD as an extension of supervised physiotherapy. The primary objective of this study is to evaluate the feasibility and potential efficacy of Reality DTx^®^.

**Methods:**

Twenty-four pwPD (Hoehn and Yahr stages 2–4) with self-reported gait and/or balance impairments will participate in this study. The study comprises a 6-week waitlist-controlled AR home-based therapeutic gait-and-balance exergaming intervention. Reality DTx^®^ will initially be prescribed remotely for a minimum of 5 days a week for 30 min per day. We will remotely set and adjust the frequency, difficulty, type of games, and/or duration weekly, based on objective and subjective data from the AR glasses and participant, respectively. In addition to the home-based gait-and-balance exergaming intervention, the study comprises three laboratory visits: before the 6-week waitlist period (t0; baseline), before the 6-week intervention period (t1; pre-intervention), and after the 6-week intervention period (t2; post-intervention). The primary study parameters are feasibility (in terms of safety, adherence, and user experience) and potential efficacy for improving gait and balance (using standard clinical gait-and-balance tests and a targeted walking-related fall-risk assessment). Recruitment started in December 2022 and the final post-intervention assessment will be according to planning in July 2023.

**Conclusions:**

This clinical feasibility trial is the first remotely prescribed and monitored home-based AR gait-and-balance exergaming intervention for pwPD. The results in terms of clinical feasibility (i.e. safety, adherence, and user experience) and potential efficacy (gait, balance, and fall-risk outcomes) form the basis for future randomised controlled studies on the effectiveness of home-based AR gait-and-balance exergaming interventions for pwPD.

**Trial registration:**

ClinicalTrials.gov, NCT05605249. Registered on 4 November 2022.

**Supplementary Information:**

The online version contains supplementary material available at 10.1186/s40814-024-01480-w.

## Background

For people with Parkinson’s disease (pwPD), gait-and-balance impairments are common and disabling. Gait impairments are varied across pwPD but are typically continuous in nature (shuffling, slow, and asymmetrical gait pattern) and can also become episodic when the disease progresses (i.e. freezing of gait) [1, 2]. Balance impairments often lead to falls related to retropulsion or stumbling, a relative inability to make sufficiently large compensatory balance-correction steps and alter body position effectively [2–4]. These motor symptoms are related to a loss of independence and a decreased quality of life [2, 5, 6].

Clinical guidelines addressing gait-and-balance impairments stress that, alongside pharmacological treatment, physiotherapy and exercise should be given a central role in disease management [7–11]. Exercise can be described as a planned, structured, repetitive, and purposeful physical activity to maintain one or more components of physical fitness, such as strength or balance [10, 12]. Different forms of exercise, ranging from isolated training of specific motor skills to multifaceted exercise like dance or boxing, have repeatedly been shown to improve both motor and non-motor symptoms [6, 12–17]. Current clinical guidelines agree that exercise interventions for pwPD should target multiple components, including gait and balance, endurance, strength, flexibility, and functional-based training like getting up from a chair [7–11], and be specifically designed for pwPD to address their motor symptoms [17].

**Fig. 1:**
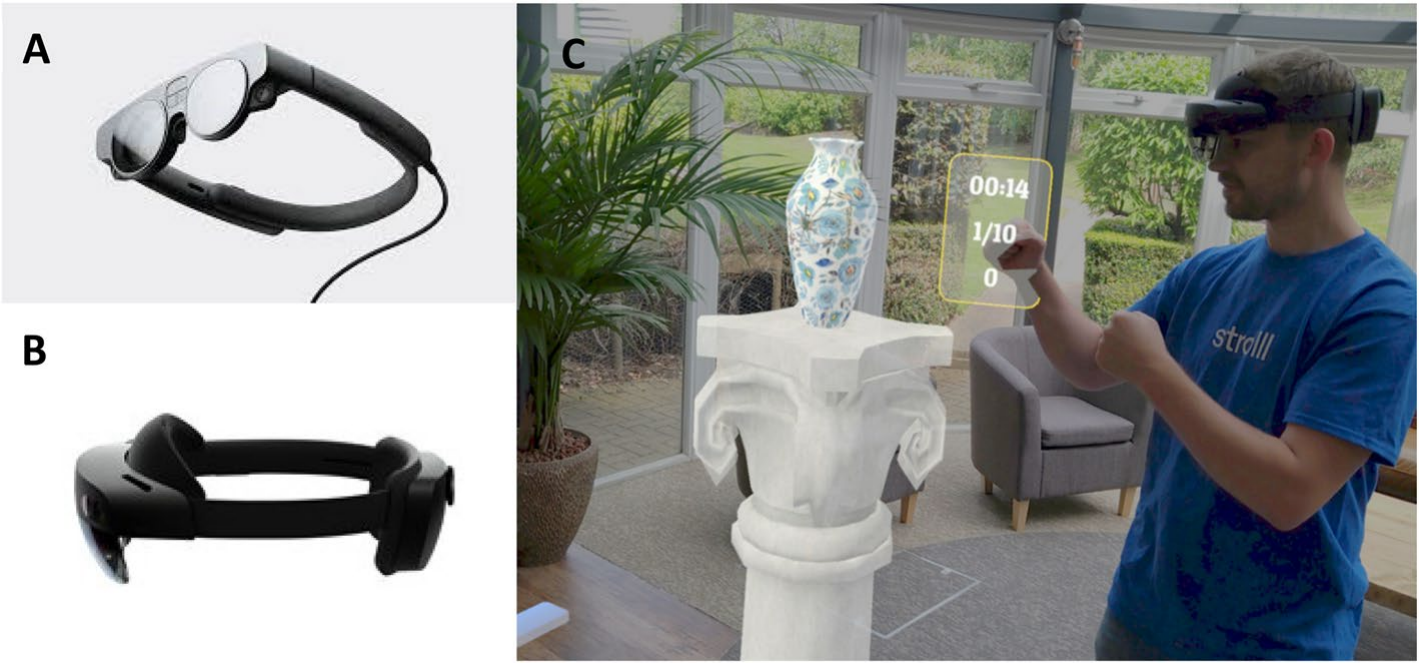
Augmented-reality (AR) glasses (Magic Leap 2 (**A**) and HoloLens 2 (**B**)) for performing gait-and-balance exercises at home using Reality DTx^®^ therapeutic exergames. In (**C**), ‘Smash’ is illustrated, an AR boxing game promoting weight shifts, dynamic balance, turning, and walking with direct feedback on task duration, number of required punches, and number of smashed holograms like the depicted vase)

The augmented-reality (AR) exercise intervention proposed in this clinical feasibility study protocol is specifically designed to address gait-and-balance impairments of pwPD and does that in a personalised and accessible manner. This is much needed as doing regular exercise remains a challenge for pwPD. A way to promote gait-and-balance exercise and increase adherence is to provide a personalised gait-and-balance intervention [18–20] according to the FITT principles [21] that offers exercises at the right Frequency, Intensity, of the right Type and Time (i.e. duration). Novel supporting technologies, like AR glasses, may be exploited to increase adherence by (i) providing individually tailored treatment (e.g. through the FITT principles), (ii) allowing for (online) remote monitoring of therapy adherence and performance (e.g. when delivered in a home setting), (iii) making treatment more accesible (e.g. available at any time when delivered in a home setting) and (iv) motivating users through play and instant rewarding feedback [22–25]. AR (sometimes referred to as ‘mixed reality’) is an immersive technology that merges real and digital worlds. Through AR glasses, like Magic Leap and Microsoft’s HoloLens (Fig. 1A and B), the real world can be augmented with spatial-aware digital objects (a.k.a. holograms) while—in contrast to virtual-reality (VR)—the real world remains fully visible via see-through lenses (Fig. 1C). The HoloLens has already been successfully and safely used previously in pwPD to target goal-directed movement with visual cues [26, 27]. In light of the potential of AR as an immersive technology to deliver, personalise, monitor, and promote exercises remotely at home, Reality DTx^®^ was developed by Strolll Limited [28].

Reality DTx^®^ is specifically designed for pwPD to improve their gait and balance, both in clinic and at home, with AR exercises being gamified, personalised, and accessible (at any time) to maximise adherence. Hence, Reality DTx^®^ can be regarded as AR therapeutic exergaming intervention, a combination of gait-and-balance exercises and gaming delivered through wearable AR glasses. Taking into account the low adherence to prescribed exercises by PD physiotherapists in standard care [20], a home-based AR gait-and-balance exergaming intervention like Reality DTx^®^ might improve the ease of accessibility to therapeutic exercises and therefore increase exercise adherence, help achieving the prescribed required number of repetitions, and potentially its effect through personalised interventions (e.g. following FITT principles), motivational feedback, and remote monitoring by the therapist.

In this clinical feasibility study, we evaluate a 6-week remotely prescribed and monitored home-based AR gait-and-balance exergaming intervention for pwPD (Reality DTx^®^). The primary objective is to evaluate its feasibility (in terms of safety, adherence, and user experience [usability and acceptability]) and potential efficacy for improving gait and balance (in terms of standard clinical and laboratory-based gait-and-balance test outcomes and targeted walking-related fall-risk indicators). The secondary objective is to examine AR glasses’ superiority (i.e. potential differences between Magic Leap 2 and HoloLens 2 subgroups) in those regards. In this study protocol, we outline the methods used to reach both objectives and discuss potential implications for future research and clinical practice.

## Methods

### Trial design

This clinical feasibility trial protocol is designed as a waitlist-controlled 6-week home-based AR gait-and-balance exergaming intervention, in which the type of AR glasses (Magic Leap 2, HoloLens 2) will be counterbalanced over participants (Table 1). All participants will start with a 6-week waitlist period, followed by the 6-week home-based AR therapeutic gait-and-balance exergaming intervention Reality DTx^®^. Baseline (t0), pre-intervention (t1), and post-intervention (t2) repeated measurements will be conducted to compare within-participant Reality DTx^®^ intervention effects against usual care (i.e. does t2–t1 differ from t1–t0?) while controlling for potential learning effects in outcomes over repeated measurements (i.e. does t1–t0 systematically deviate from 0?). Between-group comparisons will be performed to evaluate potential AR glasses superiority (i.e. does t2–t1 differ between HoloLens 2 and Magic Leap 2 subgroups)? Feasibility of the Reality DTx^®^ intervention will be examined in terms of safety, adherence, and user experience (i.e. usability and acceptability), including an evaluation of AR glasses superiority in that regard.

**Table 1:**
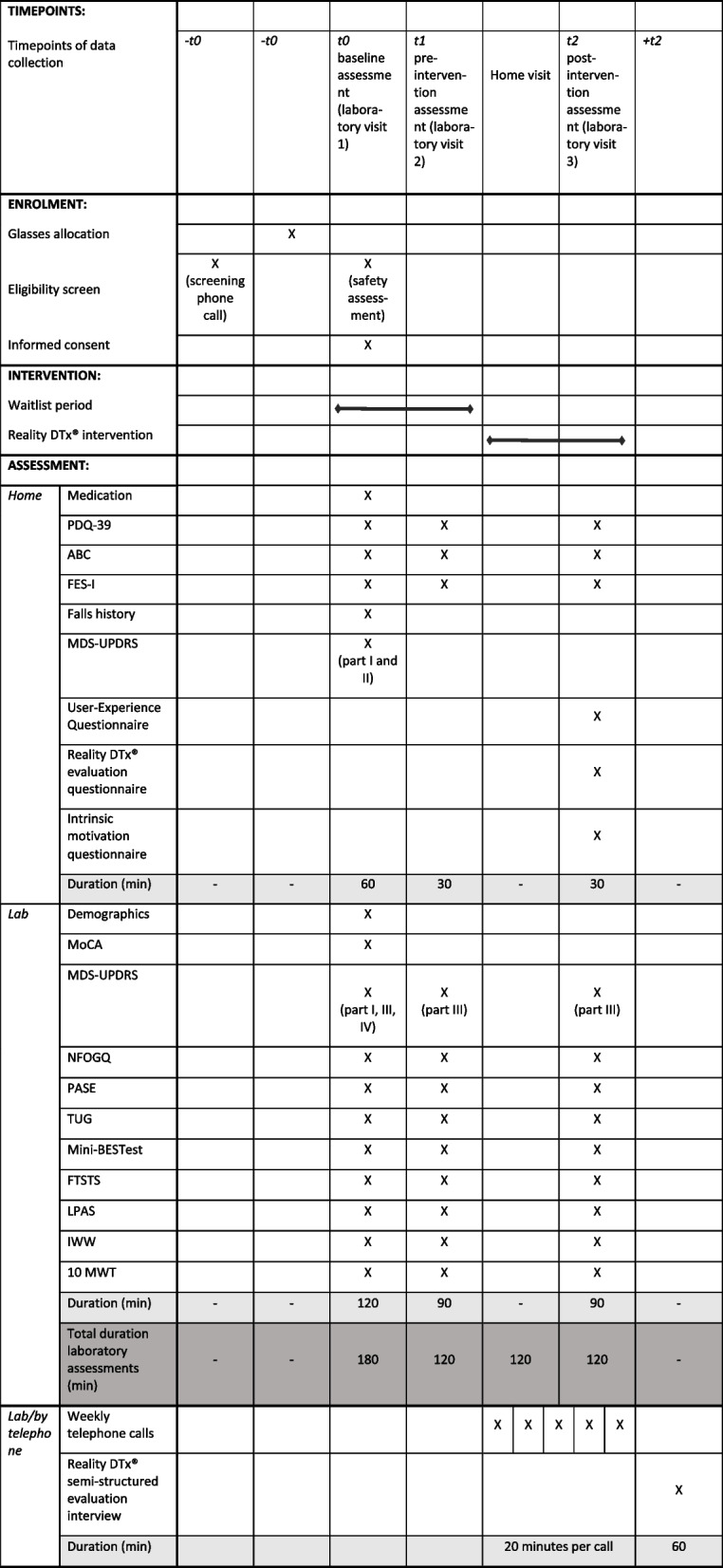
Illustrates the time points, enrolment, waitlist and intervention period, and the (duration of) assessments

### Intervention

Reality DTx^®^, a class I CE marked medical device, is a software application for AR glasses HoloLens 2 and Magic Leap 2. In the proposed study, participants will follow a 6-week AR therapeutic exergaming intervention (Reality DTx^®^) comprising five complementary gait-and-balance exercises, as detailed in Table 2 (see Additional file 6 for videos of the gait-and-balance exergames). In accordance with the clinical guidelines [7–11], participants will initially be invited to use Reality DTx^®^ for 30 min per day for a minimum of 5 days a week, in their home environment. Participants are instructed that they are allowed to train more if they wish.

**Table 2:**
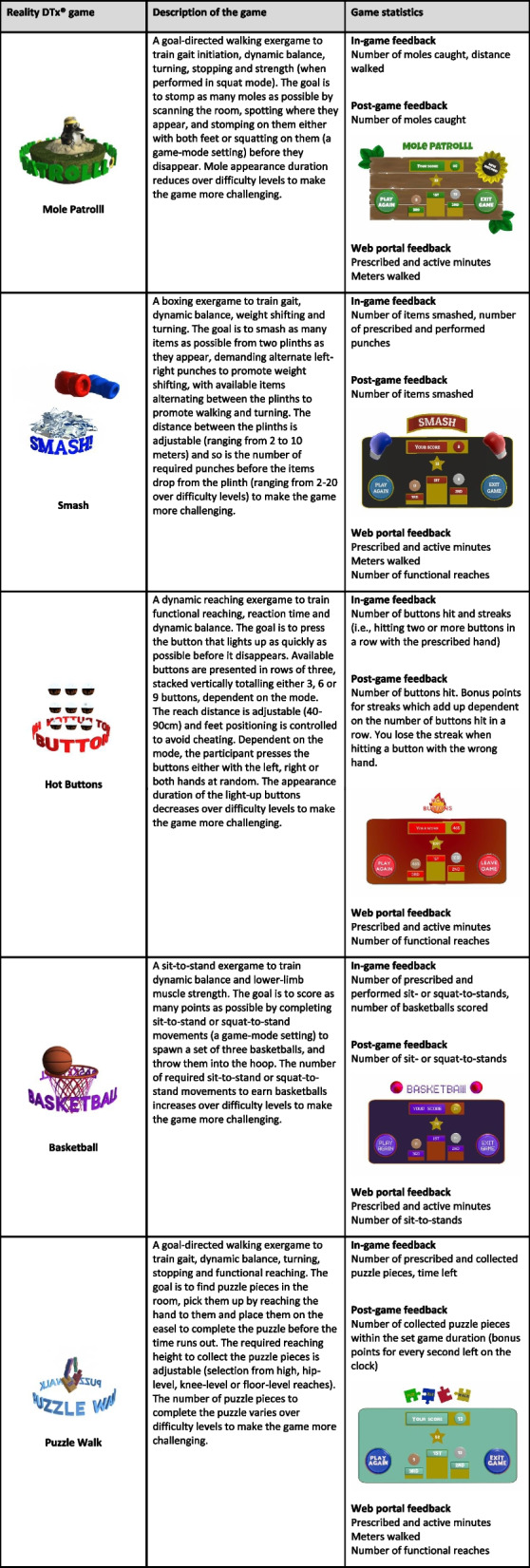
Description of the five AR gait-and-balance Reality DTx^®^ exergames, including available game statistics

All five gait-and-balance games will initially be set at a 3-min duration and participants are instructed to complete them twice. The initial difficulty levels and game modes (see Table 2) will be adjusted to the participants’ gait-and-balance competence levels, as evaluated at t0 and t1. The exergaming schedule will be further personalised on a weekly basis applying shared decision-making. That is, personalisation is based on remotely monitored therapeutic exergame adherence and performance scores as well as participant-reported feedback from semi-structured weekly telephone calls (e.g. enquiring about adherence, performance, and (serious) adverse events (AE), including (near) falls and potential physical problems, as detailed below and in Additional file 3). This will result in a personalised remotely prescribed gait-and-balance exergame schedule for the next week for which frequency (number of prescribed therapeutic exergame sessions per week), intensity (by varying the difficulty level and/or game mode; see Table 2), type (type of game), and/or time (duration per game and/or therapy session) may be varied. The personalised exergaming schedule will be accessible for the participant through the AR glasses at any time during the day. Participants are free to play all prescribed gait-and-balance games in a single session or to divide them into so-called exercise snacks over the day. After finishing the prescribed exergaming schedule on a day, participants unlock bonus ‘exercise snacks’ in the so-called free-play mode, enabling participants to perform additional AR gait-and-balance exergames of their liking if they wish to. Participants receive direct feedback on gait-and-balance exergame performance during the game (e.g. number of moles hit, number of items smashed, number of buttons hit; see Table 2) and total scores upon game completion (i.e. in relation to personal high scores).

### Procedure

Duration: 12 weeks.

Study setting: Gait laboratory at the Department of Human Movement Sciences of Vrije Universiteit Amsterdam (three visits, baseline [t0], pre-intervention [t1], and post-intervention [t2]) and participants’ home environments (one home visit to set up the Wi-Fi connection for the AR glasses to be able to remotely monitor and prescribe gait-and-balance exergames, to select a safe exergaming space in the home and link this to the AR glasses and to instruct the participant how to operate, charge, and store the AR glasses, followed by 6-week independent but remotely monitored exergaming with weekly telephone calls to personalise remotely prescribed exergaming schedules).

**Fig. 2:**
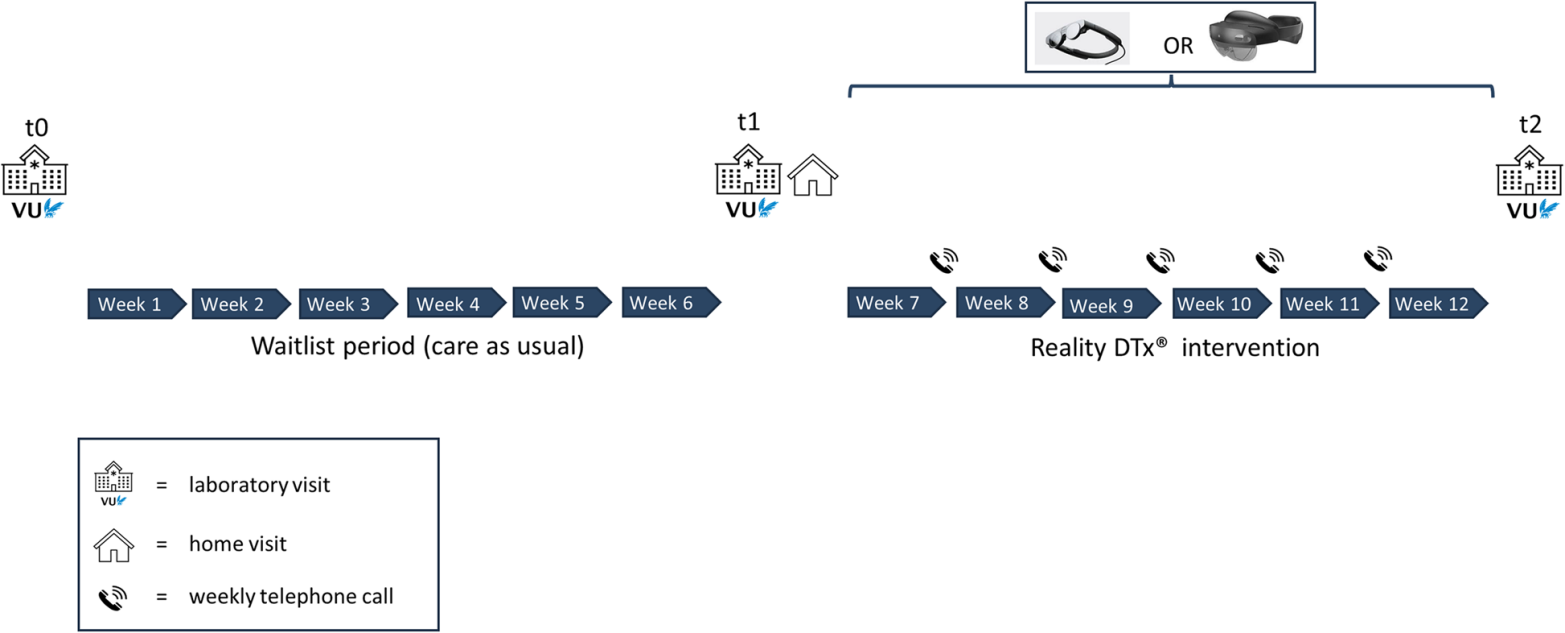
Overview of the study design with the three laboratory assessments (t0, t1, t2), the 6-week waitlist-control and Reality DTx^®^ intervention periods, the counterbalancing of AR glasses over participants, and the weekly telephone calls to personalise participants’ gait-and-balance exergaming schedule

As visualised in Fig. 2, the feasibility trial comprises:

- three visits to the gait laboratory to evaluate potential intervention effects vis-à-vis potential learning effects in outcomes over repeated measurements during the waitlist-control period (first laboratory visit: baseline assessment [t0], second laboratory visit: pre-intervention assessment [t1], and the third laboratory visit: post-intervention assessment [t2]),
- one home visit to set up the AR glasses for independent but remotely monitored use
- five semi-structured telephone calls (as detailed in Additional file 3) to enquire about adherence, performance, and safety (including (serious) adverse events in relevant classes [29, 30] using questions like ‘Did you fall at any time during the training this week?’, ‘Did you nearly fall at any time during the training this week?’, ‘Did you experience any physical problems during training this week, such as dizziness, eye strain, headache or something else …’), usability (including technical issues) and perceived usefulness of the intervention, as well as to decide on the gait-and-balance exergaming schedule for the subsequent week in collaboration with the user (i.e. shared decision-making).

In between gait-laboratory visits 1 and 2 (i.e. t0 and t1, delineating the 6-week waitlist-control period), participants will not receive any instructions or training regarding Reality DTx^®^ and will carry out their activities and care as usual. The Reality DTx^®^ AR therapeutic exergaming intervention is intended as an intervention additional to usual care. Therefore, there will be no restrictions to care as usual during the 6-week intervention period, except for changing the dosage of medication (see exclusion criteria). Usual care and potential changes therein will be reported over the 6-week waitlist-control period as well as over the 6-week Reality DTx^®^ intervention period. In between laboratory visits 2 and 3 (i.e. t1 and t2, delineating the 6-week Reality DTx^®^ intervention period), participants will independently use Reality DTx^®^, remotely monitored and prescribed personalised AR gait-and-balance therapeutic exergaming in their home environment.

#### Laboratory assessments (t0, t1, t2)

Table 1 provides an overview of the data collected during the baseline (t0), pre-intervention (t1), and post-intervention (t2) laboratory assessments, which include demographic data, questionnaire data, and standard clinical gait-and-balance test data. Participants will also undergo a gait assessment on the Interactive Walkway (IWW), a validated instrumented 10-m walkway for markerless full-body 3D motion registration [31–35] to assess gait parameters during a standard 10-m walk test (e.g. walking speed, step length, cadence) and to perform a targeted walking-related fall-risk assessment, focusing on walking-adaptability tasks using projections on the walkway to elicit step or gait adjustments for precision stepping, sudden turning, sudden obstacle avoidance, and narrow-beam walking [32, 34, 35]. The order of the gait-and-balance tests (i.e. TUG, FTSTS, Mini-BESTest, and IWW) will be counterbalanced over participants but remains fixed per participant over t0, t1, and t2 laboratory visits.

During baseline (t0) and pre-intervention (t1) assessments, participants will get the opportunity to practice and familiarise themselves with the Reality DTx^®^ gait-and-balance exergames. After the post-intervention assessment (t2), participants will be asked to participate in a semi-structured scripted on-site or telephone interview (as detailed in Additional file 5) to evaluate Reality DTx^®^ therapy including various feasibility aspects regarding usability, safety, perceived efficacy, performance, context-specific factors of the training, FITT principles, the commercial potential, and to gather feedback for improvements. The interview is partly based on the theoretical framework of acceptability [36, 37]. The interview data is supplemented with existing scales for user experience [38], intrinsic motivation [39], and acceptability [40]. After this final evaluation interview, participants will receive an individual report about their performance during the Reality DTx^®^ exergaming intervention and their gait-and-balance test scores over t0, t1, and t2.

Participants are invited to participate in an optional fourth lab visit, somewhere during the intervention period, for a stand-alone experiment on the gait-modifying effects of AR cues and to assess concurrent validity and test–retest reliability of the clinical outcome measures of gait and balance derived from the AR glasses. This optional laboratory visit is beyond the scope of the feasibility study and will therefore not be addressed further in this protocol.

#### Home visit

A researcher will visit participants’ homes to set up the AR glasses (either HoloLens 2 or Magic Leap 2) and define a safe space for home-based gait-and-balance exergaming. Both AR glasses are non-occluding (see-through lenses) and use a form of simultaneous localisation and mapping (SLAM) to anchor holographic content in the real world, which allows users to control the Reality DTx^®^ software using hand tracking (e.g. pressing a holographic button) and voice commands. The AR glasses differ in weight (HoloLens 2 is untethered and weighs 566 grammes which is heavier than the Magic Leap 2 which is a tethered device (260 grammes, at the expense of a cable connecting the glasses to a waist-worn computer and battery pack)), AR field of view (double for Magic Leap), hand-tracking quality (better for HoloLens than for Magic Leap, which comes with a hand-held controller; in the proposed study, participants will be encouraged to use their hands to control Reality DTx^®^ instead of the controller), battery life (HoloLens 2–3 h, Magic Leap 3.5 h), and corrective eyewear (HoloLens can be worn over individual’s prescription glasses, for Magic Leap prescription lenses can be ordered and inserted).

During this home visit, participants will receive printed and oral instructions on how to (safely) use the AR glasses and will then do a second supervised Reality DTx^®^ gait-and-balance exergaming session with the researcher. Participants receive a diary to rate their experience after every exergaming session on a 5-point Likert scale. They will also be asked to administer the exercise day and how many times they played each therapeutic exergame. Potential falls [41] and technical issues are also noted. Participants may write down any points they want to discuss during the weekly telephone call (see Additional file 2 for details on the diary).

#### Remote monitoring of gait-and-balance exergaming adherence and performance and telephone calls to personalise the remote prescription of exergaming schedules

**Fig. 3:**
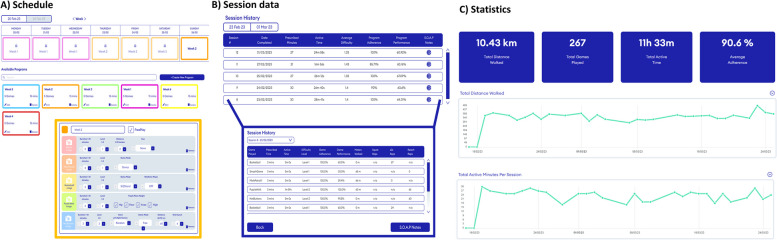
Snapshots of the web portal to remotely prescribe (**A**) and monitor (**B **and **C**) gait-and-balance exergames. Please see Table 2 for a description of all adjustable Reality DTx^®^ gait-and-balance exergaming elements per game

The Reality DTx^®^ web portal is designed by Strolll as an online e-portal for healthcare professionals to prescribe, monitor, and adjust the Reality DTx^®^ gait-and-balance exergaming intervention and to remotely track the participant’s progress (Fig. 3). In the proposed study, the web portal will be used to remotely prescribe personalised exergaming schedules on a weekly basis (Fig. 3A) and to monitor the participant’s adherence and performance remotely (Fig. 3B, C). The initial training schedule (frequency, difficulty, type, and duration) is created by the researchers based on the supervised Reality DTx^®^ familiarisation and practice sessions during the laboratory visits (t0, t1) and home visit. The training schedule will be adjusted by the researcher every week through the Reality DTx^®^ web portal. Adjustments are made in close collaboration with the participants based on their subjective experiences evaluated during the weekly semi-structured telephone call (see Additional file 3) and based on the (objective) adherence (performed gait-and-balance exergames as a percentage of the prescribed gait-and-balance exergames) and performance (the score per game) scores available in the Reality DTx^®^ web portal. The weekly telephone calls will also be used to ask if participants experienced any technical issues (usability flags) or adverse events (safety flags).

### Participants and recruitment

We aim to recruit 24 participants with PD. Recruitment started in December 2022. According to planning, the final post-intervention assessment (t2) will be in July 2023. Participants will be recruited using various channels: through regular clinical care (i.e. via neurologists and physiotherapists in our network) and via presentations at various Parkinson community groups hosted by the Dutch Parkinson Association and through the website of the Dutch Parkinson Association. People who are interested in participating will receive detailed written information about the study before consenting to participate in this study. The information letter was drafted in consultation with patient representatives of the Dutch Parkinson Association and approved by the medical ethical committee. At least 1 week after receiving the information letter, potential participants will be contacted through telephone calls by the researchers to check whether they understood the information letter and to answer any questions. After affirming that a potential participant is willing to participate, the following eligibility criteria will be checked.

#### Inclusion criteria

To be eligible to participate in this study, participants must meet the following criteria: 18 years or older, have command of the Dutch language, diagnosed with PD according to the UK PD Brain Bank criteria (stages 2–4 on the Hoehn and Yahr scale), and experience bothersome gait and/or balance impairments based on self-report (i.e. negatively affecting their ability to perform their usual daily activities).

#### Exclusion criteria

Potential participants will be excluded from participation in this study when meeting any of the following criteria: inability to comply with the protocol, i.e. additional neurological diseases and/or orthopaedic problems seriously interfering with gait-and-balance function, insufficient physical capacity or cognitive/communicative inability to understand instructions and participate in the tests (as observed by the researchers), visual or hearing impairments (after corrective aids), severe visual hallucinations or illusions, inability to walk independently for 30 min, and no stable dosages of dopaminergic medication.

After passing the telephonic eligibility screening, participants will be invited to the gait laboratory where they will sign for written informed consent (see Additional file 1) before the start of the baseline assessment. They will be informed explicitly about the possibility to be excluded from the study after baseline based on an objective assessment of the eligibility criteria and/or serious safety concerns. To this end, participants’ demographics will be documented to characterise the study population in terms of age, gender, disease duration, current medication use (type, dose, and frequency), and scores on Movement Disorders Society Unified Parkinson Disease Rating Scale (MDS-UPDRS), Montreal Cognitive Assessment (MoCA) and falls history. Safety and eligibility concerns will be based on performed motor/cognitive/falls-risk assessments as well as a supervised Reality DTx^®^ gait-and-balance exergaming session during t0. The entire baseline assessment session will be recorded on video to discuss participants’ safety and eligibility concerns among the researchers, who will then jointly decide on exclusion or not. Reasons for potential exclusion will be documented as part of the feasibility evaluation.

### Randomisation, blinding, and treatment allocation

In this waitlist-controlled clinical feasibility study, all participants will receive Reality DTx^®^, a home-based remotely monitored and prescribed personalised AR gait-and-balance therapeutic exergaming intervention. The intervention will be delivered through two different types of AR glasses (i.e. HoloLens 2 or Magic Leap 2), which will be distributed over participants using block randomisation in blocks of four and at the end of recruitment in blocks of two to increase the likelihood for equal groups given the potential exclusion of participants after the baseline assessment and potential unforeseen recruitment issues (executed by LH and EH). Hence, half of the participants will receive the Reality DTx^®^ intervention on HoloLens 2 and the other half on Magic Leap 2. The participants and researchers will not be blinded for the allocated AR glasses.

### Sample size

For the proposed study, in which Reality DTx^®^ therapeutic gait-and-balance exergaming will for the first time be used independently at home, we will include a convenience sample of 24 participants to evaluate its initial feasibility (safety, adherence, and user experience) and potential efficacy for improving gait and balance. This sample size fits this type of study and is logistically feasible given the associated number of laboratory visits (72), weekly telephone calls (120), and home visits (24) vis-à-vis the availability of the gait laboratory and personnel, project duration, and funding. In addition, a sample of 24 participants is sufficient to evaluate the potential efficacy of this exergaming intervention. That is, with 95% power, a one-tailed alpha error of 5% and an expected minimum improvement of 1.63 s on the Timed Up-and-Go test (i.e. smallest detectable difference [42], a sample size of 18 is required to detect an effect with an effect size of 0.815 (Cohen’s *d* statistic; SD over repeated measurements 2.0 s [43]). This a priori required sample size calculation for differences between two dependent means was calculated with G*Power 3.1.9.7.

### Outcomes

#### Primary study parameter

The primary study parameters to evaluate the home-based AR gait-and-balance exergaming intervention are feasibility and potential efficacy:

Feasibility is expressed in:

- Safety, i.e. the number of (serious) adverse events categorised as falls, near falls, dizziness, eyestrain, headache, or other [29, 30] associated with the exergaming intervention, as administered during weekly semi-structured telephone calls and the Reality DTx^®^ evaluation interview. A fall is defined as a slip or trip in which one loses balance and lands on the floor, ground, or lower level [41]. A near fall is defined as a slip, trip, or loss of balance that would result in a fall if adequate recovery mechanisms were not activated [41].
- Adherence, i.e. compliance to the prescribed personalised exergaming intervention as measured in frequency (ratio of performed to prescribed number of exergaming sessions), time (i.e. duration, the ratio of performed to prescribed exergaming minutes per session), and repetitions (number of performed in-game functional motor tasks like number of squats, metres walked, number of functional reaches, …) as recorded in the web portal on a weekly basis throughout the 6-week Reality DTx^®^ intervention period, as well as the number of dropout participants, including reason(s) for withdrawal if specified,
- User experience, i.e. (1) usability based on the Dutch 26-item User-Experience Questionnaire [38] administered at t2, (2) the number and nature of the reported technological issues (e.g. AR glasses, software, Wi-Fi) during the 6-week intervention period, (3) acceptability based on a previously used intervention evaluation Likert-scale questionnaire [40] (as specified in Additional file 4) at t2, and (4) a semi-structured evaluation interview (as described previously and specified in Additional file 5) at t2 [36, 37].

Potential efficacy of the Reality DTx^®^ intervention will be explored using gait-and-balance outcome measures from the following standard clinical and IWW gait-and-balance tests, all assessed at all three timepoints (t0, t1, and t2, see Table 1):

- Balance: Mini Balance Evaluation Systems Test [44],
- Functional mobility: Timed Up-and-Go test (s) [45],
- Gait mobility: Lindop Parkinson’s Physiotherapy Assessment Scale [46],
- Walking adaptability: Targeted walking-related fall-risk assessment based on outcome measures of walking adaptability as determined with the IWW (obstacle avoidance margins and success rates and stepping accuracy and walking speed during goal-directed stepping) [31–35].

#### Secondary study parameters

Secondary study parameters include additional gait-and-balance outcome measures and patient-reported outcome or experience measures (PROMs/PREMs) to further evaluate the feasibility and potential efficacy, assessed online or in the laboratory at three time points (t0, t1, and t2, see Table 1):

- Lower limb strength: Five Times Sit to Stand Test [47],
- Motor disease severity: Scores of Movement Disorders Society Unified Parkinson Disease Rating Scale (MDS-UPDRS) [48],
- Presence of freezing of gait: New Freezing of Gait Questionnaire [49],
- Balance confidence: Activities-Specific Balance Confidence Scale [50],
- Fear of falling: Falls Efficacy Scale International [51],
- Physical activity: Physical Activity Scale for the Elderly [52],
- Quality of life: Parkinson’s Disease Questionnaire [53],

assessed online after the 6-week Reality DTx^®^ intervention period:

- Intrinsic motivation: Intrinsic Motivation Inventory [39, 54].

### Discontinuation or modification of allocated intervention

Participants can leave the study at any time for any reason if they wish to do so without any consequences. The investigator can decide to withdraw a participant from the study for urgent medical reasons or when medication dosage is changed during the study. Participants who drop out or who withdraw before commencing the intervention (that is, before the home visit) will be replaced by a newly recruited participant. Reasons for dropout or withdrawal will be collected. In case of unforeseen circumstances such as illness, technical issues or holidays that interrupt the planned training period, and if the participant is physically able and willing to resume the training once the unforeseen circumstance has been resolved, the training period will be extended to compensate for the missed days.

### Premature termination of the study

The study will be terminated prematurely if serious adverse events (SAE, like injurious falls) related to the Reality DTx^®^ intervention are reported for more than two participants. Liability insurance is in place in accordance with the legal requirements in The Netherlands, specifically Article 7 of the Medical Research Involving Human Subjects Act (in Dutch: Wet Medisch-wetenschappelijk Onderzoek met Mensen, WMO). This insurance provides cover for damage to research participants through injury or death caused by the study. The insurance applies to the damage that becomes apparent during the study or within 4 years after the end of the study.

### Statistical analysis

Data analysis will be performed in JASP [55]. Missing data will be excluded analysis-by-analysis. The Reality DTx^®^ semi-structured evaluation interview will be analysed qualitatively.

#### Feasibility of Reality DTx^®^

Study parameters to evaluate clinical feasibility (safety, adherence, user experience) of the Reality DTx^®^ intervention will be compared between the two groups (HoloLens 2, Magic Leap 2) using independent-sample *t*-tests. The Shapiro–Wilk test will be used to check for normality. If the data is not normally distributed, the Mann–Whitney *U*-test will be used. Weekly scores of feasibility parameters (e.g. adherence scores over the intervention period) will undergo 2 (between-subjects factor Group: HoloLens 2 vs Magic Leap 2) × 6 (within-subjects factor Weeks: week 1 to 6) mixed ANOVA.

#### Potential efficacy of Reality DTx^®^

The study parameters to evaluate potential efficacy of the Reality DTx^®^ intervention will be subjected to a 2 × 3 mixed ANOVA with the within-subject factor Time (three levels: t0, t1, and t2) and the between-subjects factor Group (two levels: HoloLens 2, Magic Leap 2). The assumption of sphericity will be checked according to Girden [56]. If Greenhouse–Geisser’s epsilon exceeds 0.75, the Huynh–Feldt correction will be applied; otherwise, the Greenhouse–Geisser correction will be used. Effect sizes will be quantified with ${{\eta }_{p}}^{2}$.

Paired-sample *t*-tests will be used for post hoc comparisons of significant main and/or interaction effects involving the factor Time (or paired-sample Wilcoxon tests if data is not normally distributed according to the Shapiro–Wilk test). We expect no main effect of Group, nor a Time by Group interaction, but only a main effect of Time, with superior performance after the Reality DTx^®^ intervention (t2) than before (t0, t1). In case t1 differs significantly from t0 as well (which may suggest learning/habituation in repeated test performance), an additional test on difference scores will then be performed to compare the magnitudes of intervention (t2–t1) and learning/habituation (t1–t0) effects with a paired-samples *t*-test (or paired-samples Wilcoxon tests if data is not normally distributed according to the Shapiro–Wilk test). We expect that potential intervention effects will be greater in magnitude than potential learning/habitation effects.

Because this is a feasibility trial, these comparisons will also be evaluated with Bayesian hypothesis testing [57, 58] using JASP [55], quantifying how much more likely the data support the alternative hypothesis (gait-and-balance outcomes differ over Time or Groups) compared to the null-hypothesis (gait-and-balance outcomes do not differ over Time or Groups), reported as the Bayes factor BF_10_ (alternative/null). In line with Jeffreys [57], we regard BF_10_-values between 1 and 3 as anecdotal evidence, values between 3 and 10 as moderate evidence, and values above 10 as strong evidence for the alternative hypotheses.

## Discussion

This is the first remotely prescribed and monitored, personalised home-based AR therapeutic exergaming intervention specifically designed for pwPD to address their gait-and-balance impairments. The primary objective is to assess its feasibility (is it safe, adherable, accepted, and usable?) and to explore its potential efficacy (does it improve gait-and-balance outcomes?). The secondary objective is to evaluate AR glasses’ superiority in those regards (does it matter which AR glasses are used for performing, monitoring, and prescribing gait-and-balance exergaming?).

### Improving gait-and-balance exergaming with AR

To date, research into exergaming in Parkinson’s rehabilitation almost primarily focused on interventions presented on so-called non-immersive devices (e.g. Xbox Kinect or Nintendo Wii) [22, 59–63]. In several recent reviews, non-immersive exergaming interventions were considered at least equivalent in effectiveness for improving gait and balance and strengthening the effects of traditional supervised physiotherapy when combined [59–62]. A recent systematic review on home-based exergaming interventions concluded that exergaming in a home setting is effective in improving balance, mobility, and gait outcomes. Moreover, adherence to home-based exergaming was high, operationalised in terms of observance, enjoyment, and number of dropouts [22]. Therapeutic exergaming thus has strong potential for improving gait and balance. This form of delivering exercise may be particularly well suited for pwPD given that they are less inclined to engage in exercise due to motor symptoms (e.g. cardinal symptoms affecting range of motion) as well as non-motor symptoms (e.g. apathy, fatigue, anxiety and fear of falling) that reduce physical activity [21, 64, 65].

In the proposed clinical feasibility study, immersive AR technology will for the first time be used to deliver interactive exergaming for improving gait and balance in pwPD. One obvious advantage of AR exergaming is that one can directly interact with the digital content (i.e. physically step onto a digital mole popping out a molehill visible on the floor in the real world): there is no separation between the real and the digital world, allowing for task-specific training of visuomotor control of gait and balance. This stands in stark contrast with non-immersive or VR exergaming technologies where the interaction between movements made in the real world and the presented digital content is typically indirect (i.e. one can position a visual-feedback representation of a step made in the real world towards a mole popping out a molehill displayed on a screen.: Because of this indirect visuomotor coupling, the required magnitude of movements made in the real world to position a digital visual-feedback representation on a digital target has to be learned. Considering the reliance on visual (augmented) feedback and attention for motor control in pwPD [66, 67], exergaming with direct interactions in immersive environments (in contrast to indirect interactions in non-immersive or VR environments) might enhance perceptual-motor learning by directing attention and vision to task-relevant digital objects in the real world, that might, akin to real-world objects, act as affordances (i.e. possibilities) for action [66, 68, 69].

### Safety of home-based AR exergaming

A key objective of clinical feasibility trials is to address safety and adverse events of novel interventions like the AR-supported gait-and-balance therapeutic exergaming intervention Reality DTx^®^. This is deemed especially important when the exergaming intervention is delivered in an unsupervised home setting in a high fall-risk population like ours. Although several systematic reviews have reported that non-immersive exergaming to train gait and balance in pwPD is safe for use in both rehabilitation and home settings [22, 59, 60, 62], some adverse events in pwPD have been reported with both non-immersive (a non-injurious fall in a home-based step training study [70]) and immersive exergaming interventions (eyestrain and minor motion sickness during a dancing intervention using Google Glass [71]). We do not expect many adverse effects in the form of eyestrain and motion sickness, which is quite common in VR but not or less so with AR, in the proposed feasibility study. That is, Reality DTx^®^ runs on state-of-the-art AR glasses, where interactive augmented-reality content naturally blends with the real world and is largely static in case of the proposed study [29, 30]. Nevertheless, safety of use of immersive AR exergaming in the home setting of pwPD is yet to be determined.

To maximise safety of the participants in the current clinical feasibility study, the following recommendations have been implemented: (1) assess safety by the researchers (one of which is a trained clinician) during the first two laboratory assessments as well as via weekly semi-structured telephone calls during the 6-week intervention, (2) provide instructions on safety of use of the gait-and-balance exergames during the first two laboratory assessments and the home visit prior to the 6-weeks of home-based AR gait-and-balance exergaming, and (3) to tailor and adjust the gait-and-balance exergames to the functional level of the participant using remotely monitored objective information about adherence and performance as well as subjective information from weekly semi-structured telephone calls [22, 24, 62, 72].

### Future steps after this feasibility trial

The results on feasibility and potential efficacy will identify methodological challenges for future randomised controlled trials [73] and form the basis for design choices (e.g. required sample size, primary outcome measures) regarding the effectiveness of home-based AR gait-and-balance interventions for pwPD. Specifically, the complementary selection of outcome measures, quantifying various aspects of gait and balance such as walking adaptability, dynamic balance, and strength, will give a comprehensive insight into the specific constructs tackled with Reality DTx^®^ exergaming while the scores from baseline, pre-intervention, and post-intervention assessments will provide indications of obtainable effect sizes. Together, this will inform about the most specific and sensitive outcome measures to demonstrate gait-and-balance effects with remotely monitored and prescribed home-based AR therapeutic exergaming.

Such future studies will contribute to solving a societally important problem: accessibility to treatment. PD is a growing disease, and the number of pwPD is expected to double in the next two decades. This will increase the burden on already understaffed care, resulting in longer waitlists and suboptimal treatment. Technology like Reality DTx^®^ to safely deliver effective treatment (partly) at home may help counter this doom scenario, allowing pwPD to complete personalised and monitored therapeutic gait-and-balance exercises of the right frequency, intensity, with high levels of repetition, type, and duration in the convenience of their own home (saving time, costs, and burden associated with travelling to the healthcare professional) and own time (exercising whenever they feel ready, taking into account fatigue, medication effects and matters of work, household, and family), while potentially saving the healthcare system time and costs (the same healthcare professional can treat more pwPD). Being able to remotely monitor adherence and performance of the gait-and-balance exercises is crucial to guard the quality of treatment and to personalise treatment frequency, intensity, type, and time. With the pwPD participating in this feasibility trial, we will evaluate safety, adherence, acceptability, and usability of such a new care model supported by AR technology while future randomised controlled trials should address its effect and cost-effectiveness.

### Supplementary Information

**Additional file 1. **Informed consent. The informed consent format to be signed by the study participants.

**Additional file 2. **Participant diary. A diary provided to the participant at the start of the 6-week intervention period to be filled in after every exergaming session.

**Additional file 3. **Weekly phone call script. Semi-structured questions on adherence, performance, perceived usefulness, usability, potential technical issues, and adverse events.

**Additional file 4. **Reality DTx^®^ evaluation questionnaire. Likert-scale questions to evaluate the acceptability of the intervention.

**Additional file 5. **Reality DTx^®^ semi-structured evaluation interview script. Semi-structured interview questions addressing the participant’s experiences with Reality DTx^®^.

**Additional file 6. **Videos of Reality DTx^®^ gait-and-balance exergames. Examples of participants performing the Reality DTx^®^ games during a laboratory visit.

**Additional file 7. **SPIRIT checklist.

## Data Availability

The results of this study will be published in international peer-reviewed journals and will be presented at national as well as international scientific conferences, Parkinson association meetings, and social media through professional channels. Anonymised research data will be published along with articles as supplemental material or will be made available in a repository and/or from the corresponding author on reasonable request and with permission of Strolll Limited for any Reality DTx^®^ data that fall under contractual agreement between Vrije Universiteit Amsterdam and Strolll Limited.

## Abbreviations

pwPD: People with Parkinson’s disease
AR: Augmented-reality
VR: Virtual-reality
PROMs: Patient-reported outcome measures
PREMS: Patient-reported experience measures
IWW: Interactive Walkway
FITT: Frequency, Intensity, of the right Type and Time (i.e. duration) of an exercise programme
SLAM: Simultaneous localisation and mapping
WMO: Medical Research Involving Human Subjects Act; in Dutch: Wet Medisch-wetenschappelijk Onderzoek met Mensen
AE: Any undesirable experience occurring to a participant during the study, whether or not considered related to the medical device
SAE: A serious adverse event is any untoward medical occurrence or effect that: results in death, is life-threatening (at the time of the event), requires hospitalisation or prolongation of existing in patients’ hospitalisation, results in persistent or significant disability or incapacity, is a congenital anomaly or birth defect; or any other important medical event that did not result in any of the outcomes listed above due to medical or surgical intervention but could have been based upon appropriate judgement by the investigator. An elective hospital admission will not be considered a serious adverse event
