# Supplementary material for: Remotely prescribed and monitored home-based gait-and-balance therapeutic exergaming using augmented reality (AR) glasses: protocol for a clinical feasibility study in people with Parkinson’s disease
Source: Pilot Feasibility Stud. 2024 Mar 27;10:54. doi: 10.1186/s40814-024-01480-w (PMC10967163; doi:10.1186/s40814-024-01480-w)
Supplement: Supplementary file 1 — Additional file 1. Informed consent. The informed consent format to be signed by the study participants. [file 40814_2024_1480_MOESM1_ESM.docx]

**Informed consent form for research participants**Related to:
Reality DTx®: Home-based training for improving gait and balance

Team Holocue(X), Vrije Universiteit Amsterdam

- I have read the information letter and had the opportunity to ask questions. My questions have been adequately answered, and I had sufficient time to decide whether I want to participate.
- I understand that participation is voluntary, and I may withdraw my participation at any time without giving a reason.
- I am aware that I can revoke my consent for the use of my personal data without any negative consequences for myself as a participant.
- I authorize the researcher to inform my general practitioner that I am participating in this study.
- I consent to the researchers collecting and using my data, such as medication use, symptoms, and outcomes of standard gait and balance tests, only to answer the research question of this study (i.e., to determine the feasibility and effect of Reality DTx®).
- I understand that some individuals may have access to all my personal data for study monitoring purposes. These individuals are listed in the information letter, and I grant them permission to access my data for this purpose.
- Please mark "yes" or "no" in the table below:

| I consent to the recording of video footage to determine the baseline level of the Reality DTx® training program. | Yes ☐ | No ☐ |
| --- | --- | --- |
| I consent to the anonymous sharing of my movement and environmental data from the smart glasses with Strolll for further development of Reality DTx®. | Yes ☐ | No ☐ |
| I consent to the storage of my data for future research as described in the information letter. | Yes ☐ | No ☐ |
| I consent to being contacted for potential participation in future studies after the completion of this study. | Yes ☐ | No ☐ |

- By signing below, I indicate that I wish to participate in this study.

## My name is (participant): ……………………………….. Signature: ……………………… Date: __ / __ / __

-------------------------------------------------------------------------------------------------------------------------------------------------
I confirm that I have fully informed the participant about the study described above. If any information emerges during the study that may affect the participant's willingness to participate, I will inform them in a timely manner.

Researcher's name (or representative): ……………………………….
Signature: ……………………… Date: __ / __ / __
