# Supplementary material for: Remotely prescribed and monitored home-based gait-and-balance therapeutic exergaming using augmented reality (AR) glasses: protocol for a clinical feasibility study in people with Parkinson’s disease
Source: Pilot Feasibility Stud. 2024 Mar 27;10:54. doi: 10.1186/s40814-024-01480-w (PMC10967163; doi:10.1186/s40814-024-01480-w)
Supplement: Supplementary file 2 — Additional file 2. Participant diary. A diary provided to the participant at the start of the 6-week intervention period to be filled in after every exergaming session. [file 40814_2024_1480_MOESM2_ESM.docx]

Week: **Reality DTx®** Do you need help? You can reach us on: 020-5981274

| **Day** | **Did you train?** | **How often did you play every game?** | | | | | **Did you fall during training?** | **How did the training go?** | **Did you experience technical problems with the glasses?** | **Notes:** |
| --- | --- | --- | --- | --- | --- | --- | --- | --- | --- | --- |
|  | | **Mole Patrolll** | **Smash** | **Basketballl** | **Puzzle Walk** | **Hot Buttons** |  | | | |
| **Friday** | YES / NO |  |  |  |  |  | YES / NO | 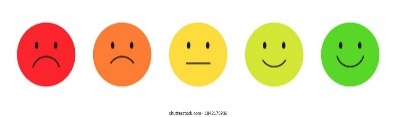 | YES / NO |  |
| **Saturday** | YES / NO |  |  |  |  |  | YES / NO | 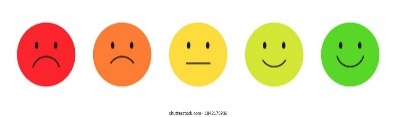 | YES / NO |  |
| **Sunday** | YES / NO |  |  |  |  |  | YES / NO | 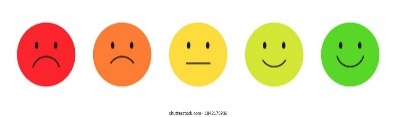 | YES / NO |  |
| **Monday** | YES / NO |  |  |  |  |  | YES / NO | 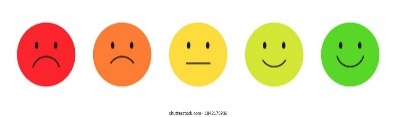 | YES / NO |  |
| **Tuesday** | YES / NO |  |  |  |  |  | YES / NO | 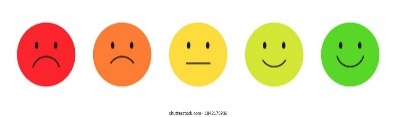 | YES / NO |  |
| **Wednesday** | YES / NO |  |  |  |  |  | YES / NO | 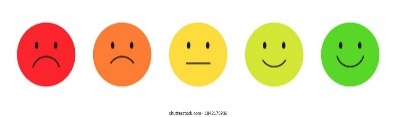 | YES / NO |  |
| **Thursday** | YES / NO |  |  |  |  |  | YES / NO | 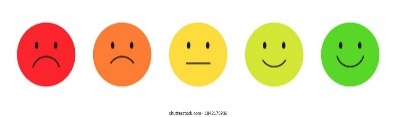 | YES / NO |  |

**What would you like to discuss during the weekly phone call? ________________________________________________________________________________________________________________________________________________________________________________________________________________________________________________________________________________________________________________________________________________________________________________________________________________________________________________________________________________________________________________________________________________________________________________________________________________________________________________________________________________________________________________________________________________________________________________________________________________________________________________________________________________________________**
