# Supplementary material for: Remotely prescribed and monitored home-based gait-and-balance therapeutic exergaming using augmented reality (AR) glasses: protocol for a clinical feasibility study in people with Parkinson’s disease
Source: Pilot Feasibility Stud. 2024 Mar 27;10:54. doi: 10.1186/s40814-024-01480-w (PMC10967163; doi:10.1186/s40814-024-01480-w)
Supplement: Supplementary file 3 — Additional file 3. Weekly phone call script. Semi-structured questions on adherence, performance, perceived usefulness, usability, potential technical issues, and adverse events. [file 40814_2024_1480_MOESM3_ESM.docx]

- *How did the training go this week?*
- **Adherence**: *In the web portal I could see that you trained* ***[adherence in terms of frequency and duration].*** *What were your experiences on adhering to the prescribed training program?*
  If the participant did not manage to train as much as prescribed, ask the participant why:
  - - Technical issues?
    - Physical capacity?
    - Time?
    - …
- **Performance**: Go through the game statistics in the web portal. Discuss every game separately and fill in the table below. Ask: *Was* ***[name of the game]*** *too easy, too difficult or something in between? Was* ***[name of the game]*** *(still) challenging enough?* Explain that you could change the difficulty of every game (by increasing the level or mode), the type of games in the program and the duration of every game or the frequency and duration for the training program as a whole. In close collaboration with the participant, decide on the training program for the following week based on: the experienced difficulty, whether the games were challenging enough and the adherence (Is the duration and frequency feasible?).

| **Game** | **Easy** | **Average** | **Difficult** | **Changes to the game settings (difficulty, type or duration) or training program as a whole (frequency, duration) for next week?** | **Notes** |
| --- | --- | --- | --- | --- | --- |
| Hot buttons |  |  |  |  |  |
| Mole Patrolll |  |  |  |  |  |
| Puzzle walk |  |  |  |  |  |
| Basketball |  |  |  |  |  |
| Smash |  |  |  |  |  |

- **Safety**:
  *Did you fall* at any time during the training this week?
  Did you nearly fall** at any time during the training this week?*
  *a fall includes a slip or trip in which one lost balance and landed on the floor or ground or lower level (41).
  ** a near fall includes a slip, trip, or loss of balance that would result in a fall if adequate recovery mechanisms were not activated (41).
- **Technical problems***:* *Did you experience any technical problems with the glasses this week?*
- **Adverse events**: *Did you experience any physical problems during training this week, such as: dizziness, eye strain, headache or something else, which is: …*
- **Usability (in week 2/4):** *On a scale from 0 to 10, how useful do you find the training? On a scale from 0 to 10, how user friendly do you find the glasses/Reality DTx® application?*
- *Do you have any questions or remarks that you would like to share?*
