# Supplementary material for: Remotely prescribed and monitored home-based gait-and-balance therapeutic exergaming using augmented reality (AR) glasses: protocol for a clinical feasibility study in people with Parkinson’s disease
Source: Pilot Feasibility Stud. 2024 Mar 27;10:54. doi: 10.1186/s40814-024-01480-w (PMC10967163; doi:10.1186/s40814-024-01480-w)
Supplement: Supplementary file 4 — Additional file 4. Reality DTx® evaluation questionnaire. Likert-scale questions to evaluate the acceptability of the intervention. [file 40814_2024_1480_MOESM4_ESM.docx]

**Reality DTx® evaluation questionnaire (online)**
The following statements are about the Reality DTx® training. This is the 6-week home-based training program with the glasses that you completed. For each of the statements about the Reality DTx® training, please provide an answer by moving the slider between the numbers 0 and 10 or the percentages 0 and 100 (question 7 and 8). For example: if you move the slider closer to ‘totally disagree', it means that you disagree with the statement more than you agree.

1. I think the Reality DTx® training was useful.

| 0 | 1 | 2 | 3 | 4 | 5 | 6 | 7 | 8 | 9 | 10  **Totally agree** |
| --- | --- | --- | --- | --- | --- | --- | --- | --- | --- | --- |

Could you elaborate on that?

**Totally disagree**

______________________________________________________________________________________________________________________________________________________________________________________________________________________________________________________

1. I think the Reality DTx® training was motivating.

| 0 | 1 | 2 | 3 | 4 | 5 | 6 | 7 | 8 | 9 | 10  **Totally agree** |
| --- | --- | --- | --- | --- | --- | --- | --- | --- | --- | --- |

**Totally disagree**

Could you elaborate on that?

______________________________________________________________________________________________________________________________________________________________________________________________________________________________________________________

1. I think the Reality DTx® training was challenging.

| 0 | 1 | 2 | 3 | 4 | 5 | 6 | 7 | 8 | 9 | 10  **Totally agree** |
| --- | --- | --- | --- | --- | --- | --- | --- | --- | --- | --- |

**Totally disagree**

Could you elaborate on that?

______________________________________________________________________________________________________________________________________________________________________________________________________________________________________________________

1. I think the Reality DTx® training was fun.

**Totally disagree**

**NO**

| 0 | 1 | 2 | 3 | 4 | 5 | 6 | 7 | 8 | 9 | 10  **Totally agree** |
| --- | --- | --- | --- | --- | --- | --- | --- | --- | --- | --- |

Could you elaborate on that?

______________________________________________________________________________________________________________________________________________________________________________________________________________________________________________________

1. I think the Reality DTx® training was user friendly.

| 0 | 1 | 2 | 3 | 4 | 5 | 6 | 7 | 8 | 9 | 10  **Totally agree** |
| --- | --- | --- | --- | --- | --- | --- | --- | --- | --- | --- |

Could you elaborate on that?

**Totally disagree**

______________________________________________________________________________________________________________________________________________________________________________________________________________________________________________________

1. I think the Reality DTx® training was suitable for improving gait and balance.

| 0 | 1 | 2 | 3 | 4 | 5 | 6 | 7 | 8 | 9 | 10  **Totally agree** |
| --- | --- | --- | --- | --- | --- | --- | --- | --- | --- | --- |

**Totally disagree**

Could you elaborate on that?

______________________________________________________________________________________________________________________________________________________________________________________________________________________________________________________

| 0 | 10 | 20 | 30 | 40 | 50 | 60 | 70 | 80 | 90 | 100  **Very likely** |
| --- | --- | --- | --- | --- | --- | --- | --- | --- | --- | --- |

1. How likely are you to recommend Reality DTx® to a friend (with Parkinson)?

**Very unlikely**

| 0 | 10 | 20 | 30 | 40 | 50 | 60 | 70 | 80 | 90 | 100  **Very likely** |
| --- | --- | --- | --- | --- | --- | --- | --- | --- | --- | --- |

How likely is it that you would continue training with this device if you could keep it?

**Very unlikely**

How would you feel if we would stop developing Reality DTx®?

Very disappointed

Somewhat disappointed

Not disappointed
