# Supplementary material for: Remotely prescribed and monitored home-based gait-and-balance therapeutic exergaming using augmented reality (AR) glasses: protocol for a clinical feasibility study in people with Parkinson’s disease
Source: Pilot Feasibility Stud. 2024 Mar 27;10:54. doi: 10.1186/s40814-024-01480-w (PMC10967163; doi:10.1186/s40814-024-01480-w)
Supplement: Supplementary file 5 — Additional file 5. Reality DTx® semi-structured evaluation interview script. Semi-structured interview questions addressing the participant’s experiences with Reality DTx®. [file 40814_2024_1480_MOESM5_ESM.docx]

**Semi-structured interview script Reality DTx®**

This interview guide provides an overview of the semi-structured questions per interview topic. The interviewer used follow-up questions to obtain more detailed information regarding the individual experiences of the participants.

Introduction to participant:

- This interview is about the 6-week home-based training program with Reality DTx®.
- Results of the lab visit tests will be shared with you after the interview.
- There are no right or wrong answers. We value all participants honest opinions to improve the Reality DTx® intervention.
- Feel free to add anything you would like to share but is not asked.

**GENERAL EXPERIENCE:**

1. Can you think of one word to describe your experiences with the Reality DTx® intervention?

Please can you explain why you have chosen this word?

1. Affective attitude (36): How do you feel about the training? What made you feel this way?

**EFFECTIVENESS:**

1. Perceived effectiveness (36): To what extent did you experience effects from the training?
2. Intervention coherence (36): Can you explain to me, in your own words, what the goal of the training was?

A. If not mentioned yet: Did you experience improvement in walking or balance
 during or after the 6-week training period?

- Yes
- No

If yes, what kind of improvements?

B. If not mentioned yet: Did you experience improvement in performing any daily life activities?

- Yes
- No

If yes, what kind of improvements?

1. Ethicality (36): To what extent did the training fit your views on home-based gait and balance exercise?
2. What type of people do you think would most benefit from Reality DTx®?

**PERFORMANCE**:

1. Self-efficacy (36): how did you do in the training?
   Did you become more confident in playing the games during the training?

**FITT:**

1. What did the participant think of the Reality DTx® intervention, in terms of:
2. Frequency/duration:
   A1. What did you think of the duration of one training session (i.e., 30 minutes)?
   - Too short
   - Short
   - Good
   - Long
   - Too long

Can you please elaborate on that?

A2. What did you think of the duration of the training program as a whole? (i.e., 6
weeks)

- - Too short
  - Short
  - Good
  - Long
  - Too long

Can you please elaborate on that?

A3. What did you think of the frequency of the training program? (i.e., 5 days a week)

- - Too infrequent
  - infrequent
  - Good
  - often
  - Too often

Can you please elaborate on that?

A4. Opportunity costs (36): To what extent did you have to give up on other activities or values to participate in the training?

1. Burden (36): What did you think of the difficulty of the training (in general, not per game)?

- Too easy
- Easy
- Good
- Hard
- Too hard

Can you please elaborate on that?

1. Intensity: What did you think of the intensity of the training?

- Too light
- Light
- Good
- Hard
- Too hard

Can you please elaborate on that?

1. Do you think your physical fitness has increased?

- Yes
- No

Can you please elaborate on that? What did you notice?

1. Type: What did you think of the type of training? (e.g. gait and balance, type of exercises)

What game did you like the most? What game did you like the least?

**USABILITY:**

1. What was your initial attitude about training with this headset and what about now?
2. Weight: What did you think about the weight of the glasses?
3. Field of view: What did you think about the visibility of the holograms in the games?
4. Controlling the holograms:

Handtracking: what did you think about controlling the holograms/buttons in the game with your hand?
If you could chose, how would you like to control/select the buttons/holograms (explain every option briefly)?:

- - Voice command (by giving a voice command to select a button/hologram)
  - Position in space (by standing in the same position as the button/hologram)
  - Hands
  - Eyes (by looking at the button/hologram)

1. Wearing comfort

How long do you think you could wear the glasses before it becomes uncomfortable?
…. hour(s) … min

1. Magic Leap 2 versus Hololens 2

Magic Leap 2:
What did you think about the headset?
What would you change about the headset?
Did you find the battery pack obstructive?

Hololens 2:

What did you think about the headset?

What would you change about the headset?

**ADVERSE EVENTS/SAFETY:**

1. Did you experience any difficulties in terms of eye sight during Reality DTx® training? That is, were all letters and holograms clearly visible and readable?
2. *Did you experience any of the following problems during the last week of training: eye strain, dizziness, headache, something else, which is: …?
3. *On a scale from 0 to 10, how useful did you find the training? On a scale from 0 to 10, how user friendly did you find the glasses/Reality DTx® application?
4. Did you feel unsafe at any point during the Reality DTx® training?

- Yes
- No
  Can you please elaborate on that?

1. *Did you fall/nearly fall during the last week of training?
   *a fall includes a slip or trip in which one lost balance and landed on the floor or ground or lower level (40).
   a near fall includes a slip, trip, or loss of balance that would result in a fall if adequate recovery mechanisms were not activated (40).*
2. *Did you experience any technical difficulties during the last week of training?

* This question is asked as part of the final weekly phone call (week 6).

**CONTEXT-SPECIFIC FACTORS (36):**

1. What did you think about training individually?
2. What did you think about training at home?
3. What did you think about the scoring system that was used during training?
4. What did you think about remote supervision?

**COMMERCIAL POTENTIAL:**

1. If you would need to pay a monthly prescription for the headset (which includes the headset, the application and technical support), what would you pay?
2. Would you prefer traditional physiotherapy (including home exercises prescribed as usual) or would you prefer fewer physiotherapy sessions (e.g., every 3 weeks) but supplemented with the remotely monitored (by the physiotherapist) Reality DTx® home-based exercise intervention?

**IMPROVEMENTS REALITY DTX®:**

A. Do you have any suggestions for improving the Reality DTx® training program?

B. If you could give a suggestion, what improvement or new feature should we tackle/develop first?
